# Protective role of resveratrol against VCM-induced hepatotoxicity in male wistar rats

**DOI:** 10.3389/fphar.2023.1130670

**Published:** 2023-02-07

**Authors:** Fahad S. Alshehri, Nasser M. Alorfi

**Affiliations:** Department of Pharmacology and Toxicology, College of Pharmacy, Umm Al-Qura University, Makkah, Saudi Arabia

**Keywords:** vancomycin, resveratrol, hepatotoxicity, hepatoprotection, glycopeptide

## Abstract

**Background:** Vancomycin is a glycopeptide antibiotic with a high risk of acute liver injury. Resveratrol is believed to protect the liver against toxicity.

**Aim:** To investigate the ability of resveratrol to attenuate vancomycin-induced liver toxicity in rats injected with vancomycin.

**Method:** Twenty-four adult male Wistar rats were distributed into three groups. The control group received only a vehicle, while the treated group received either vancomycin 200 (mg/kg, i. p.) only or vancomycin (200 mg/kg, i. p.) with resveratrol (20 mg/kg, oral gavage). All groups received their dose once daily for 7 days. Hepatic damage was assessed by measuring biochemical parameter levels in serum, aspartate transaminase (AST), alanine transaminase (ALT), alkaline phosphatase (ALP), and lactate dehydrogenase (LDH). Also, antioxidants and inflammation biomarkers such as Interleukin-6 (IL-6), malondialdehyde (MDA), nitric oxide (NO), and glutathione (GSH) were measured. Furthermore, the vancomycin-induced pathological changes in the liver were evaluated by histopathological studies.

**Results:** In the vancomycin-treated group, hepatic serum biomarkers such as AST, ALT, ALP, IL-6, and MDA were elevated, while NO and GSH were depleted. However, resveratrol co-treatment with vancomycin prevented the elevation of AST, ALT, ALP, IL-6, and MDA and it protected the liver from NO and GSH depletion. Also, regarding vancomycin-induced degeneration of hepatocytes, resveratrol co-treatment with vancomycin prevented such degeneration and improved mononuclear cells in the liver.

**Conclusion:** The results showed that oral administration of resveratrol has a significant hepatoprotective effect against vancomycin-induced hepatotoxicity.

## Introduction

Many drugs are known to produce liver injury, and these adverse hepatic events usually result in severe liver injury if not treated properly (Bissell et al., 2001). It has been estimated that drug-induced liver failure represents half of the cases of all forms of acute and chronic liver disease (Kaplowitz, 2001). Approximately 10% of chronic hepatitis cases occur due to drug use, and 5% from hospital admissions, while 50% of acute liver failure cases occur due to drug use (Pandit et al., 2012). Hepatotoxicity associated with antibiotics is asymptomatic and usually presents mild hepatic injury (Thiim and Friedman, 2003). Vancomycin is a glycopeptide antibiotic with known bactericidal activity, and it is considered the drug of choice for treating methicillin-resistant *Staphylococcus aureus* infections (David and Daum, 2010; Steinmetz et al., 2015). However, several side effects have been reported with vancomycin, such as hypotension, phlebitis, nephrotoxicity, and hepatotoxicity (Badran et al., 2011; Bamgbola, 2016).

Moreover, a few reports have shown that chronic use of glycopeptide antibiotics has the potential to elevate liver enzymes and induce hepatotoxicity (Cadle et al., 2006; Chen et al., 2011; Brunetti et al., 2020). However, data to support the influence of vancomycin on liver dysfunction are limited, and the mechanism of vancomycin-induced hepatotoxicity has not been studied effectively. Many risk factors contribute to vancomycin-induced hepatotoxicities, such as long-term treatments, high doses, obesity, patient age, and overall health (Larrey, 2002; Breedt et al., 2005; Kohno et al., 2007; Florescu et al., 2008). Moreover, the hepatic injury associated with vancomycin could also be due to sepsis, bacterial endotoxins, fever, or hemolysis (Sibai, 2004; Shah et al., 2010; Kouijzer et al., 2021). While different strategies have been suggested to reduce any potential risk of hepatotoxicity associated with vancomycin treatment (Aldaz et al., 2000; Hwang et al., 2015; Regal et al., 2019; Tsutsuura et al., 2021), the exact mechanism for this injury is not fully understood. Several studies have suggested that vancomycin-induced toxicity could be due to several factors, including the generation of free radicals, oxidative stress, and inflammation, which cause liver injury in animal studies (Sahin et al., 2006; El Bohi et al., 2021). In addition, reactive oxygen species (ROS) are usually generated within cells, leading to the initiation of oxidative stress-related intermediates, which contribute to chronic inflammation and liver fibrogenesis (Bataller and Brenner, 2005; Friedman, 2008; Novo and Parola, 2008). Therefore, herbal compounds with antioxidant and anti-inflammatory properties have been considered.

Indeed, cumulative reports have suggested that herbal compounds have a great potential to attenuate drug-induced liver toxicity due to their antioxidant and anti-inflammatory properties (Abou Seif, 2016; Parthasarathy and Evan Prince, 2021). Hence, many herbal compounds have been used as traditional medicines for liver disorders (Ali et al., 2008; Zhang et al., 2018; Philips et al., 2020; Das et al., 2022). In addition, these are potential sources of new therapeutic agents that could be used to prevent hepatic injuries. For example, resveratrol has long been known to have antioxidant and anti-inflammatory effects. Moreover, researchers have recently become more interested in resveratrol, from its ability to extend human lifespans to its effect on chemoprevention, cardiovascular diseases, and neurodegenerative disorders, as reported in several studies (Gescher and Steward, 2003; Srivastava et al., 2013; Pourhanifeh et al., 2019; Banez et al., 2020; Labban et al., 2021a; Labban et al., 2021b). The antioxidant properties of resveratrol have been demonstrated in several *in vitro* studies. The antioxidant property of resveratrol has been demonstrated by inhibiting nicotinamide adenine dinucleotide phosphate oxidases, which inhibit the production of reactive oxygen species (ROS) (Halliwell, 2007; Yousefian et al., 2019). As well as protecting cells from oxidative stress, resveratrol also promotes the expression of antioxidative enzymes and their substrates (Miguel et al., 2021; Santos et al., 2021). Recently, it has been suggested that resveratrol has hepatoprotection properties through its anti-inflammatory and antioxidant effects (Chupradit et al., 2022; Ma et al., 2022; Tong et al., 2022). It has also been reported that resveratrol attenuates acetaminophen toxic metabolite N-acetyl-p-benzoquinone-imine and facilitates liver regeneration by modulating the silent mating type information regulation two homolog (SIRT1), tumor protein P53, and Tumor Necrosis Factor-alpha (TNF-α) (Sener et al., 2006; Wang et al., 2015). Moreover, resveratrol has been shown to improve glutathione (GSH) levels and antioxidant enzyme activities, and to decrease ROS production in liver tissues (Bujanda et al., 2008; Rivera et al., 2008; Rubiolo and Vega, 2008; Sebai et al., 2010). Also, one study reported that the thioacetamide-induced hepatotoxic effect associated with TNF-α and iNOS elevation was inhibited by resveratrol (Ebrahim et al., 2022).

This study investigates the effect of high doses of vancomycin administered to induce liver toxicity. A few studies have investigated similar regimens and found that high doses of vancomycin were associated with elevated levels of liver enzymes, the tissue activities of catalase, superoxide dismutase activities, lipid peroxidation, and malondialdehyde (MDA) (Ahmida, 2012; Çağlayan et al., 2019; El Bohi et al., 2021). Moreover, this research investigates the ability of resveratrol to attenuate vancomycin-induced liver toxicity through several biomarkers, such as liver tissues, inflammatory mediators, liver enzymes, and antioxidant property markers.

## Materials and methods

### Drugs

Resveratrol (ProHealth, United States) and vancomycin (Medis, Tunisia) were used in the study. All other chemicals and reagents used were of analytical grade. Resveratrol and vancomycin were dissolved in a saline solution (0.9% NaCl) as a vehicle for both drugs.

#### Dose selection

The vancomycin dose was based on several recent studies using 200 mg/kg, i. p. to induce hepatotoxicity (Kucukler et al., 2020) and nephrotoxicity (Ahmida, 2012) once daily for seven consecutive days. The resveratrol dose was based on several studies using the same dose against several compounds, such as dimethylnitrosamine (Lee et al., 2010) and concanavalin (Zhou et al., 2015).

##### Animals

Twenty-four male adult Wistar rats (weighing 170–207 g) were used. The animals were housed in plastic cages (4 rats per cage) under a 12 h light/12 h dark schedule in a humidity-controlled room and were fed a normal diet. They had access to food and water *ad libitum* and were monitored daily to ensure proper animal welfare. The rats were acclimatized for 1 week before starting the experiment. Then, the rats were distributed into three groups (n = 8 in each group). The rats received only a vehicle in the first group (control). In the second group, vancomycin, the rats received vancomycin (200 mg/kg, i. p.) once daily for seven consecutive days. In the last group, vancomycin + resveratrol, the rats received vancomycin (200 mg/kg, i. p.) and resveratrol (20 mg/kg, oral gavage) once daily for seven consecutive days. All the treatments were carried out within 7 days, and the animals were euthanized using CO_2_ and sacrificed on the eighth day. The tissue and serum samples were collected, homogenized, centrifuged, for analysis.

### Measurement of biochemical parameters

The serum samples were used for the measurement of all biochemical parameters. The usage of serum samples was based on several reportes that have useed similar methods to assess hepatic function. Aspartate transaminase (AST), Alanine transferase (ALT) as reported in (Yin et al., 2019), Alkaline phosphatase (ALP) (Ibrahim et al., 2020), Interleukin-6 (IL-6) (Xia et al., 2019), nitric oxide (NO) (Fathy et al., 2019), GSH (Ibrahim et al., 2020) and MDA (Omara et al., 2021).

#### Markers of liver tissue damage

The serum samples were analyzed using assay kits and ELISA for liver functions. Aspartate transaminase (AST), Alanine transferase (ALT) and Alkaline phosphatase (ALP) were assessed using ELISA kits (MyBioSource kits catalog: MBS269614, MBS264975, MBS011598, MBS726781; MyBioSource, Inc.) A centrifuge was then performed at approximately 1000× g for 15 min. Serum was collected, and the assay was immediately performed according to the manufacturer’s recommendations (MyBioSource, Inc.). A standard curve was established using a series diluent. A Microplate reader (450 nm detection wavelength filter, 570 nm or 630 nm correction wavelength filters) was used to perform all the tests.

#### Markers of inflammation

Interleukin-6 (IL-6) and nitric oxide (NO) levels in the serum were measured with the fully automatic ELISA DSX best 2000^®^ microtiter plate and the ELISA kits. A centrifuge was then performed at approximately 1000× g for 15 min. Serum was collected, and the assay was immediately performed according to the manufacturer’s recommendations (MyBioSource, Inc.).

#### Markers of antioxidant and prooxidant

GSH and MDA levels in the serum were measured using an ELISA DSX best 2000^®^ microtiter plate and the ELISA kits. In a serum separator tube, the serum was clotted for 2 hours at room temperature and overnight at 2°C–8°C. A centrifuge was then performed at approximately 1000× g for 15 min. Serum was collected, and the assay was immediately performed according to the manufacturer’s recommendations (MyBioSource, Inc.). A Microplate reader (450 nm detection wavelength filter) was used to perform all the tests.

##### Histopathology

The liver tissues were used for the histopathological assessment and prepared in 10% formalin solution for 2 days. In addition, the tissue was embedded in paraffin blocks following routine tissue tracking procedures. Finally, Hematoxylin and eosin stains were used to stain the slides. Masson’s Trichrome method was employed, which involves deparaffinizing and rehydrating the liver in descending series of alcohols before staining them with Biebrich scarlet-acid fuchsin solution. A solution of phosphomolybdic-phosphotungstic acid was then used to differentiate the sections.

### Statistical analysis

Statistical analyses were performed using GraphPad Prism™ (v9.3.1). Data were expressed as mean ± standard error of the mean. A one-way ANOVA, followed by Tukey’s post hoc test, was used for comparisons. A *p*-value of <0.05 was considered statistically significant.

## Results

### Measured levels of AST, ALT, ALP, and LDH

**FIGURE 1:**
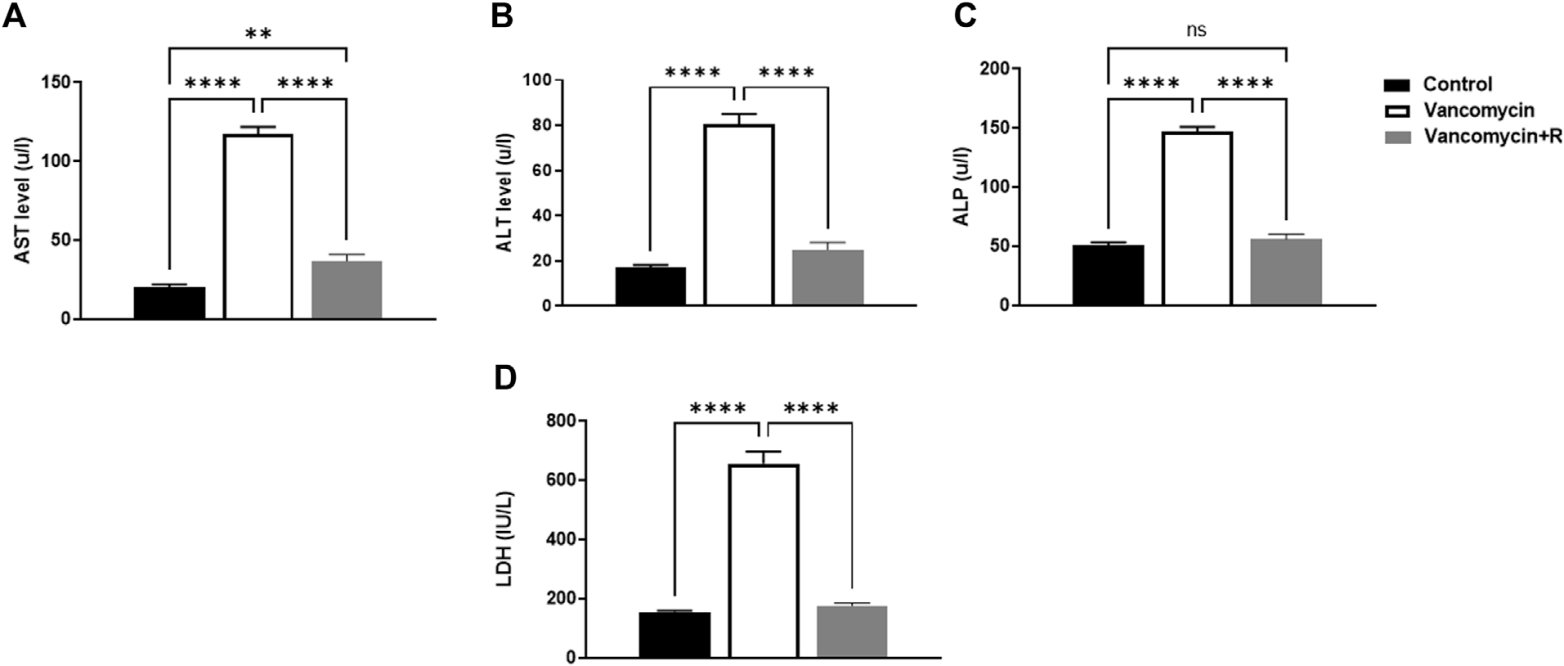
**(A)** Aspartate transaminase (AST) levels in u/l, **(B)** Alanine aminotransferase (ALT) in u/l, **(C)** Alkaline phosphatase (ALP) in unit/liter (u/l), and **(D)** lactate dehydrogenase (LDH) (IU/L), were measured in the control rats, rats injected with vancomycin only, and rats injected with vancomycin and resveratrol. Significant difference: ns = non-significant, **p < 0.001 ****p < 0.0001.

The effect of vancomycin on AST, ALT, ALP and LDH was observed in the rats’ serum. The one-way ANOVA test revealed a significant main effect on the AST serum levels [F (2, 21) = 216.0, *p* < 0.0001, Figure 1A]. Further analysis using Tukey’s multiple comparisons test revealed that rats injected with only vancomycin had significantly increased levels of AST compared to the control group (*p* < 0.0001). Interestingly, the rats injected with resveratrol and vancomycin were protected against vancomycin-induced toxicity. In addition, the vancomycin + resveratrol group of rats showed a significant increase in AST levels, *p* = 0.0083, compared to the control group.

Moreover, vancomycin had a significant main effect on the ALT serum levels in the groups [F (2, 21) = 124.0, *p* < 0.0001, Figure 1B]. Further analysis using Tukey’s multiple comparisons test revealed that the control group rats displayed no change in ALT, *p* = 0.1949. However, the rats injected with only vancomycin displayed a significant increase in ALT compared to the control group (*p* < 0.0001). Interestingly, the rats injected with resveratrol and vancomycin were protected against vancomycin-induced toxicity.

Another significant main effect on ALP serum levels in the groups [F (2, 21) = 209.3, *p* < 0.0001, Figure 1C]. Further analysis using Tukey’s multiple comparisons test revealed that the control rats displayed no change in ALP, *p* = 0.5888. The rats injected with vancomycin showed a significant increase in ALP compared to the control group (*p* < 0.0001), and the rats injected with resveratrol and vancomycin were protected against vancomycin-induced toxicity.

Moreover, significant main effect on LDH serum levels in the groups [F (2, 21) = 130.9, *p* < 0.0001, Figure 1D]. Additional analysis using Tukey’s multiple comparisons tests indicated that the control group rats had no change in LDH, *p* = 0.8200. However, the rats injected with only vancomycin displayed a significant increase in LDH compared to the control group (*p* < 0.0001). Remarkably, resveratrol demonstrated a protective role against vancomycin-induced toxicity.

### Measured levels of IL-6 and NO

**FIGURE 2:**
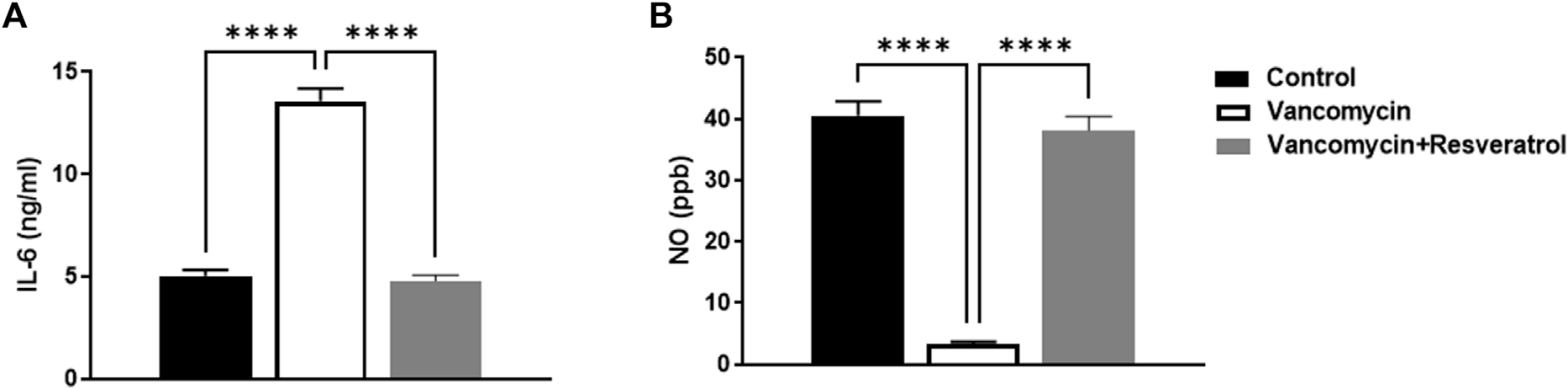
Interleukin-6 (IL-6) levels in ng/ml and **(B)** nitric oxide (NO) levels in parts per million (ppb) were measured in the control rats, rats injected with vancomycin only, and rats injected with vancomycin and resveratrol. Significant difference: ****p < 0.0001.

The effect of vancomycin on IL-6 and NO levels was observed in the rats’ serum. The one-way ANOVA test revealed that vancomycin had a significant main effect on the IL-6 serum levels in the groups F (2, 21) = 141.8, *p* < 0.0001, Figure 2A. Further analysis using Tukey’s multiple comparisons test revealed that the control rats displayed no change in IL-6, *p* = 0.9185. However, the rats injected with only vancomycin had a significant increase in IL-6 compared to the control group (*p* < 0.0001). Interestingly, the rats injected with resveratrol and vancomycin were protected against vancomycin-induced toxicity.

The significant main effect on NO serum levels in the groups [F (2, 21) = 118.3, *p* < 0.0001, Figure 2B]. Tukey’s multiple comparisons test revealed that the control group rats displayed no change in NO serum levels, *p* = 0.6613. However, the rats injected with only vancomycin showed a significant NO increase compared to the control group (*p* < 0.0001). Remarkably, the rats injected with resveratrol and vancomycin were protected against vancomycin-induced toxicity.

### Measured levels of MDA and GSH

**FIGURE 3:**
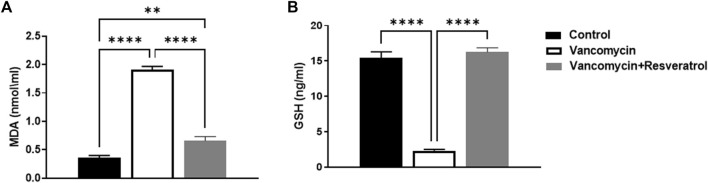
**(A)** Malondialdehyde (MDA) (nmol/ml) and **(B)** Glutathione (GSH) levels in ng/ml were measured in the control rats, rats injected with vancomycin only, and rats injected with vancomycin and resveratrol. Significant difference: **p < 0.001; ****p < 0.0001.

The effect of vancomycin on MDA was observed in the rats’ serum. A further one-way ANOVA test showed that vancomycin had a significant main effect on the MDA serum levels in the groups [F (2, 21) = 190.2, *p* < 0.0001, Figure 3A]. Also, Tukey’s multiple comparisons test revealed that the control group rats displayed a significant change in MDA serum levels, *p* < 0.0057. Again, the rats injected with only vancomycin displayed a significant increase in LDH compared to the control group (*p* < 0.0001), while the rats injected with resveratrol and vancomycin were protected against vancomycin-induced toxicity.

The effect of vancomycin on GSH levels was observed in rats’ serum. The test revealed a significant main effect on GSH serum levels in the groups [F (2, 21) = 167.3 < 0.0001, Figure 3B]. Further analysis using Tukey’s multiple comparisons test revealed that the control group rats displayed no change in GSH, *p* = 0.5894. However, rats injected with only vancomycin displayed a significant decrease in GSH compared to the control group (*p* < 0.0001). Interestingly, the rats injected with resveratrol and vancomycin were protected against vancomycin-induced toxicity.

#### Histopathological results

**FIGURE 4:**
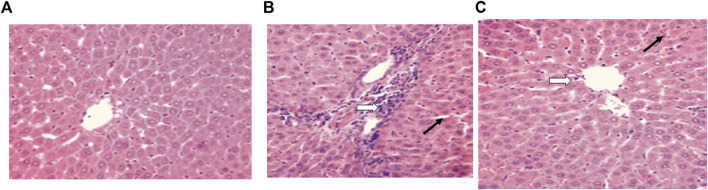
Histopathological liver evaluation in **(A)** control rats, **(B)** rats injected with vancomycin only, liver-Intralobular mononuclear inflammatory infiltrations (white arrow), and Mallory bodies (black arrow) due to degeneration of hepatocytes and increased vacuolation in the cytoplasm of hepatocytes appeared as indistinct clear vacuoles (black arrow) indicate glycogen infiltration and **(C)** rats injected with vancomycin and resveratrol, liver-showed marked improved on a mononuclear cell, and decrease number of mononuclear inflammatory infiltrates (white arrow) and decrease of Mallory bodies (black arrow).

For the control group, a microscopic examination of the liver revealed a normal appearance, as shown in Figure 4A. For the control + vancomycin group, liver-intralobular mononuclear inflammatory infiltrations and Mallory bodies were evident due to the degeneration of hepatocytes, as shown in Figure 4B. For the vancomycin + resveratrol group, the liver showed a marked improvement in the mononuclear cells, as shown in Figure 4C.

## Discussion

Biological systems depend on the liver to detoxify xenobiotics (Apte and Krishnamurthy, 2011). Several studies have shown that hepatic damage disrupts the body’s regular metabolism (Fabbrini and Magkos, 2015; Kurland et al., 2015). There are several causes of acute liver failure, including viral hepatitis, toxic liver damage caused by poisons and drugs, and ischemia (Jalan et al., 2012; Bernal and Wendon, 2013). The liver metabolises xenobiotics as the body’s first line of defense against ingested toxins and drugs, which often cause necrosis and apoptosis (Tsochatzis et al., 2014). A growing body of research focuses on the potential toxicity of antibiotics in the liver (Acevedo, 2015; Fernández et al., 2016; Zoratti et al., 2022). Vancomycin tends to cause adverse events after prolonged use, and large doses may be toxic to the liver (Kucukler et al., 2020). This study aimed to determine whether resveratrol plays a beneficial protective role against vancomycin-induced toxicity in the livers of male Wistar rats.

This study’s findings align with several other research studies that present vancomycin’s potential toxicity (Kucukler et al., 2020). However, to our knowledge, no previous study has investigated the protective role of resveratrol. This study revealed an elevation in serum biomarkers such as AST, ALT, and ALP levels in the groups given only vancomycin or vancomycin with resveratrol, compared to the control group. The serum level of ALT is the most widely used clinical biomarker of hepatic function (Senior, 2012). Furthermore, GSH, an antioxidant, was restored to the normal level in the rats injected with vancomycin and resveratrol, indicating the antioxidant activity of the latter.

Moreover, the rats injected with vancomycin only had significantly reduced GSH levels, confirming the previous findings. Several parameters were affected by the administration of vancomycin. The levels of IL-6, LDH, MDA, and NO were highly increased in the rats injected with vancomycin. Vancomycin administration caused hepatocyte damage, leading to liver enzyme elevation. Hepatotoxic studies commonly measure liver enzyme levels such as ALT, AST, and ALP as serum hepatic biomarkers for determining liver lesions (Deshpande et al., 1998; Sadeghi et al., 2008; Mehrzadi et al., 2018). In this study, vancomycin caused a significant elevation in the serum hepatic biomarkers ALT, AST, and ALP. The concentration of ALT and AST enzymes in serum reflects the severity of liver damage as these enzymes are present in high concentrations in the liver (Adeyemi and Akanji, 2011; Adeyemi and Adewumi, 2014; Yilmaz et al., 2017). In addition, many tissues in the body contain ALP; therefore, it can be considered a non-specific enzyme (López-Posadas et al., 2011). Furthermore, hepatobiliary duct dysfunction or the destruction of hepatic cell membranes can cause a rise in serum ALP, which could indicate a problem with the excretory function (Lowe et al., 2017; Kashima et al., 2018). On the other hand, co-treatment of vancomycin with resveratrol protected against vancomycin-induced hepatic damage, evidenced by the significantly decreased levels of the hepatic serums AST, ALT, and ALP.

It is well known that vancomycin is almost completely eliminated from the body by the kidneys; however, the mechanism by which nephrotoxicity occurs is still unclear. It has been demonstrated in experimental animals that the drug may cause tubular ischemia and acute tubulointerstitial injury by inducing oxidative stress in the proximal renal tubule cells (King and Smith, 2004; Gupta et al., 2011a). Here, vancomycin increased the serum levels of IL-6 (a pro-inflammatory cytokine). The cell surface receptors of the IL-6 family of cytokines regulate cell function (Taga and Kishimoto, 1997). IL-6 consists of two structural subunits: a ligand-binding subunit called the IL-6 receptor and a signal-transducing glycoprotein called Gp130 (Yamauchi-Takihara and Kishimoto, 2000). The liver synthesizes several acute phase proteins in response to IL-6 as it is involved in the pathogenesis of many fibrogenic diseases (Choi et al., 1994). In recent studies, IL-6 has been linked to acute and chronic liver damage (Cao et al., 1998; Gewiese-Rabsch et al., 2010; Bergmann et al., 2017; Shao et al., 2020). In addition, many xenobiotics drugs can injure the liver and trigger the release of pro-inflammatory cytokines like TNF-α and IL6 into the bloodstream (Takai et al., 2016; Olaniyan et al., 2018; Wu et al., 2018). By demonstrating the changes in cytokines that occur in hepatic cells, rodent models can illustrate the molecular changes associated with human hepatic cell death. In this study, we also tested the serum level of MDA, an oxidative stress biomarker that serves as an index of oxidative damage in the liver (Bakan et al., 2002). MDA has been reported to induce collagen production by hepatic stellate cells, resulting in fibrosis (Hadizadeh et al., 2017). Also, it has been reported that vancomycin could initiate an intracellular production of peroxides that triggers the production of MDA (Oktem et al., 2005). Thus, in this study, the vancomycin-induced high serum levels of MDA could be due to vancomycin’s free radical trapping activity and oxidative stress.

GSH has several functions, including serving as an antioxidant, and playing a role in redox and cell signaling (Franco and Cidlowski, 2009; Mari et al., 2009). It acts by reducing hydrogen peroxide, scavenging ROS, and reactive nitrogen species (RNS); therefore, it protects cells against oxidative damage (Day and Suzuki, 2005; Winter et al., 2017). The build-up of an oxidized form of GSH, glutathione disulfide (GSSG), and the depletion of GSH are closely related to ROS and RNS effects on the liver and cells (Yuan and Kaplowitz, 2009; Eskandari et al., 2012). Hepatic NO and its derivatives are essential in liver physiology and pathophysiology (Laskin et al., 2001; Chen et al., 2003; Diesen and Kuo, 2010). It is also a second messenger that acts in several pathways and plays a crucial role in regulating blood pressure by relaxing the endothelium, attacking tumor cells, and stimulating the brain (Gupta et al., 2011b; Korde Choudhari et al., 2013; Picon-Pages et al., 2019). Although NO has multiple and complex roles, it has been suggested that it affects the pathogenesis and progression of liver diseases (Iwakiri and Kim, 2015; Ekhlasi et al., 2017; Wang et al., 2018). On the other hand, LDH (a non-specific tissue damage biomarker) was elevated in vancomycin-treated animals. Numerous tissues and organs in the body produce LDH, including the muscles, liver, heart, pancreas, kidneys, brain, and blood. Body tissue damage can be detected by using the LDH test, determining its location and severity (Farhana and Lappin, 2022). In this study, vancomycin administration causes liver damage, contributing to LDH elevation in the serum. This elevation could arise from vancomycin, causing damage to the kidneys. It has been reported that vancomycin can cause kidney damage (Naghibi et al., 2007), making this test a non-specific marker for liver damage.

Resveratrol is a natural compound extensively studied in preclinical studies as a nutraceutical and therapeutic agent. In addition, the antioxidant properties of resveratrol have been demonstrated in a wide range of hepatic disorders (Pan et al., 2017; Bircan et al., 2018). The antioxidant effect functions mainly by reducing ROS and eliminating direct free radicals, while improving the activity of endogenous antioxidant enzymes superoxide dismutase, catalase, and GSH (Carrizzo et al., 2013; de Oliveira et al., 2018). Furthermore, it has been reported that resveratrol is involved in several vital pathways regulating *de novo* fibrogenesis deposition in the liver (Hessin et al., 2017). For example, resveratrol (10 and 20 mg/kg/day) was administered to cirrhotic rats, where it reduced portal pressure, improved vasodilatory acetylcholine responsiveness, and reduced the production of thromboxane A2, resulting in liver tissue regeneration (Di Pascoli et al., 2013; Zhang et al., 2016).

Globally, liver illnesses continue to be a severe health burden. For the treatment of this category of disorders, new and secure therapeutic options are required. This study shows that resveratrol is a good alternative in this area. This approach could significantly improve potential resveratrol therapeutic applications. Understanding how resveratrol improves many liver disease conditions may lead to novel treatment possibilities. For example, resveratrol produces beneficial effects and reduces possible toxic effects when combined with other medications and substances. As a result, there is still future work to be done because there are still significant gaps in our knowledge about this chemical.

## Data Availability

The raw data supporting the conclusions of this article will be made available by the authors, without undue reservation.
